# A cross-sectional survey of material deprivation and suicide-related ideation among Vietnamese technical interns in Japan

**DOI:** 10.3389/fpsyg.2023.1241837

**Published:** 2024-01-04

**Authors:** Tadashi Yamashita, Pham Nguyen Quy, Chika Yamada, Emi Nogami, Erina Seto-Suh, Saori Iwamoto, Kenji Kato

**Affiliations:** ^1^Faculty of Nursing, Kobe City College of Nursing, Kobe, Japan; ^2^Department of Medical Oncology, Kyoto Miniren Central Hospital, Kyoto, Japan; ^3^Department of Environmental Coexistence, Center for Southeast Asian Studies, Kyoto University, Kyoto, Japan; ^4^Department of Social Welfare, School of Psychology and Social Welfare, Mukogawa Women’s University, Nishinomiya, Japan; ^5^Human Rights Research Institute, Kindai University, Higashi osaka, Japan; ^6^Faculty of Nursing, Kobe Women’s University, Kobe, Japan

**Keywords:** Vietnam, Japan, technical intern trainees, material deprivation, suicidal ideation, COVID-19

## Abstract

**Background:**

The economic struggles faced by many technical intern trainees in Japan include the necessity to remit money to their home country, debts owed to intermediaries facilitating their arrival, and reduced working hours due to the COVID-19 pandemic. Furthermore, there is concern that the pandemic may contribute to mental instability resulting from the significant life changes experienced by the trainees. This study examined the experience of material deprivation among Vietnamese intern trainees in Japan and explored the correlation between material deprivation and suicidal ideation.

**Methods:**

A cross-sectional study was conducted between September and October 2021, involving 310 Vietnamese technical intern trainees. Data from 200 participants were analyzed. The questionnaire included gender, age, duration of residence in Japan, proficiency in the Japanese language, income changes due to the COVID-19 pandemic, material deprivation status, and suicidal ideation. Suicidal ideation was assessed using the ninth item of the Patient Health Questionnaire-9. Logistic regression analysis was performed to investigate the relationship between material deprivation items and suicidal ideation.

**Results:**

The mean age of the respondents was 26.0 ± 5.1 years, with 62.0% (*n* = 124) being male. Among the material deprivation items, 74.0% (*n* = 148) reported food deprivation, 59.0% (*n* = 118) reported cellphone bill deprivation, and 55.0% (*n* = 110) reported medical expense deprivation. Suicidal ideation was reported by 23.0% (*n* = 46) of the respondents. The prevalence of suicidal ideation was associated with age (*p* = 0.031, odds ratio [OR] = 0.889, 95% confidence interval [CI] = 0.799–0.990), deprivation of food expenses (*p* = 0.003, OR = 3.897, 95% CI = 1.597–9.511), and deprivation of cellphone usage (*p* = 0.021, OR = 3.671, 95% CI = 1.217–11.075).

**Conclusion:**

Vietnamese technical intern trainees in Japan faced various forms of material deprivation, which correlated with a high prevalence of significant psychological issues. Suicidal ideation was influenced by factors such as age, deprivation of food expenses, and inability to pay cellphone bills. The experience of material deprivation could have intensified the mental health challenges faced by Vietnamese trainees, particularly in the demanding circumstances of the COVID-19 pandemic.

## Introduction

The World Health Organization (WHO) has highlighted that the COVID-19 pandemic has resulted in a global rise in depression rates, mainly attributed to social isolation (World Health Organization, 2022). Furthermore, migrants are particularly vulnerable to mental health issues during this pandemic due to stress related to the well-being of their loved ones, financial challenges, and uncertainty about their future (World Health Organization, 2020). To promote the well-being of migrants, it is crucial to enhance their access to essential healthcare services and address linguistic and socio-cultural barriers. This is especially important in mitigating social health disparities and providing livelihood and health support to migrants who face a heightened risk of social isolation because of the COVID-19 pandemic.

By the end of 2022, the foreign resident population in Japan reached approximately 3.07 million individuals, with Vietnamese residents comprising 15.9% of the total (Immigration Services Agency of Japan, 2023). Among the various nationalities, Chinese residents constitute the largest proportion, while Vietnamese residents make up the second-largest group. Among Vietnamese residents, the most common residence status is that of technical intern, accounting for around 36% of the Vietnamese population in Japan (Immigration Services Agency of Japan, 2023). Between 2015 and 2019, the number of Vietnamese technical interns in Japan tripled. This increase can be attributed to the growing opportunities for Vietnamese trainees to work in Japanese industries and support their families in Vietnam with their earnings (Immigration Services Agency of Japan, 2020). However, in addition to the inherent challenges of working in an unfamiliar country, Vietnamese technical intern trainees encounter numerous obstacles, including significant limitations on their choice of occupation and restricted options for immigration. These factors have contributed to the physical and mental stress experienced by these trainees.

Instances of human rights violations, including the disappearance of Vietnamese technical intern trainees, verbal abuse toward migrant trainees by receiving facilities, and the dismissal of pregnant migrant trainees, have been reported in Japan (Immigration Services Agency of Japan, n.d.; Immigration Services Agency of Japan, n.d.; Immigration Services Agency of Japan, 2022). The Ministry of Justice in Japan has taken steps to prevent the recurrence of these problems. Furthermore, a previous study revealed that Vietnamese female technical intern trainees working in Japan often faced challenges in seeking healthcare and discussing their health issues, leading to deteriorated conditions (Shinohara et al., 2021). Moreover, there have been reports of financial exploitation, with intermediaries between employers and technical intern trainees in Japan overcharging for intermediary fees (Bélanger et al., 2011; Sa, 2013). These findings emphasize the significant social and health difficulties experienced by certain migrant trainees in Japan. Moreover, migrants are also at a higher risk of experiencing social deprivation (Haisken and Sinning, 2010). Studies have shown that individuals facing material deprivation are more susceptible to developing depressive symptoms and mental health disorders (Chung et al., 2018; Cheung and Chou, 2019). The COVID-19 pandemic has exacerbated the challenges faced by technical intern trainees in Japan, leading to reduced working hours, layoffs, and significant economic consequences (Tran, 2020; Hiroshima associated repository portal, 2021). In other words, Vietnamese interns are experiencing severe social deprivation due to the COVID-19 epidemic, which may lead to serious mental health problems. A cross-sectional study investigating psychological distress among 933 Vietnamese workers in Japan during the 2022 COVID-19 pandemic revealed an association between technical training residency status and factors contributing to psychological distress (Uezato et al., 2023). In Stockholm, Sweden, a study involving 10,081 individuals aged 18–29 years found that migrants, especially females, were at a higher risk of distress, with a particular emphasis on elevated risk of suicide attempts (Kosidou et al., 2012). Additionally, a Dutch study reported a higher incidence of suicide attempts among immigrant women (Burger et al., 2009). These collective findings raise concerns regarding suicidal ideation among Vietnamese apprentices in Japan during the COVID-19 pandemic.

Therefore, it is important to investigate the impact of the COVID-19 pandemic on Vietnamese trainees in Japan, particularly regarding material deprivation and suicide-related ideation. However, there is a lack of research examining the experiences of Vietnamese trainees in Japan during the pandemic in terms of material deprivation and suicide-related ideation. Therefore, this study investigates the present circumstances of material deprivation and its correlation with suicidal ideation among Vietnamese technical intern trainees.

## Methods

This study employed a cross-sectional research design and utilized an online questionnaire. The study adhered to the principles outlined in the Declaration of Helsinki and received approval from the Ethics Committee of Kobe City College of Nursing (approval number: 20124-05). Participants were provided with a detailed explanation of the study’s objectives, emphasizing voluntary participation, non-disadvantage for non-participation, and the assurance of personal anonymity. Following participants’ consent, they were enrolled in the study.

### Participants

A total of 310 responses were collected from Vietnamese technical intern trainees in Japan. For the present study, the sample size was calculated with a significance level of 0.05, power of 0.80, and an assumed incidence rate of suicidal ideation among Vietnamese at 20%. Consequently, the required sample size was determined to be 195. Considering a 60% rate of missing online questionnaire responses (Baruch and Holtom, 2008), the minimum sample size for collection was set at 312 (195 + 19.5·6). Samples with missing items in participants’ characteristics, socioeconomic status, PHQ-9, and material deprivation were excluded from the analysis. Of the 310 responses, 200, excluding those with missing items, were included in the analysis.

The web-based survey was conducted using SurveyMonkey (Momentive Inc., San Mateo, CA, United States). All instructions, questions, and answers within the survey were written in Vietnamese. Recruitment of participants was conducted by posting recruitment notices in Vietnamese community groups in Japan on Facebook (Meta Platforms Inc., Menlo Park, California, United States) during September 21 to October 21, 2021. In addition, questionnaires for recruiting participants were distributed at churches where Vietnamese gather during the same period. To ensure data integrity, the SurveyMonkey questionnaire was set to disallow duplicate responses. Eligible participants included Vietnamese nationals currently residing in Japan, aged 18 years or older. Exclusion criteria were applied to individuals who were unable to read the participation instructions or answer the questions independently. As a token of appreciation, participants who completed the survey received an online coupon worth 200 Japanese yen, sent to their registered email address.

### Measurement

The participants’ characteristics that were surveyed included age, length of residence in Japan, household composition, gender, residence status, educational background, marital status, level of Japanese language proficiency, and current medical history. The questionnaire also covered social and economic factors such as health insurance, availability of health advisors, and methods of accessing relevant information for daily life. Additionally, changes in monthly income and employment status during the COVID-19 pandemic was gathered. Furthermore, the survey aimed to capture the participants’ experiences of material deprivation since their arrival in Japan.

The respondents were asked to compare their current income with their income before the COVID-19 pandemic using a four-case method, which categorized changes as a significant decrease (decrease of 40% or more), slight decrease (decrease of 10–40%), almost the same (decrease of less than 10%), or slight increase (increase of 10–40%). Material deprivation, as defined by the Japanese Cabinet Office, refers to the lack of goods and services that are typically available and enjoyed in one’s country of residence due to economic reasons (Cabinet Office, Government of Japan, 2017) This study utilized seven items of material deprivation published by the National Institute of Population and Social Security Research; The seven items include shortages of food and clothing, difficulty paying cellphone bills, electricity bills in arrears, gas bills in arrears, rent arrears, and deprivation of medical expenses (Abe, 2014).

The material deprivation indicator assessed respondents’ experiences of being unable to pay for or purchase goods while in Japan. Respondents were provided with four response options: never, rarely, sometimes, or often (Saunders and Abe, 2010; Abe, 2014). To measure the degree of clinical symptoms of depression, the Patient Health Questionnaire-9 (PHQ-9) was utilized. The PHQ-9 consists of four levels (not at all, several days, more than half of days, and nearly every day) and is designed to assess the severity of depressive symptoms (Spitzer et al., 2014). The Vietnamese version of the PHQ-9 was employed in this study, with previous studies (Nguyen et al., 2016; Pham et al., 2019) validating its internal reliability, yielding a Cronbach’s alpha coefficient of 0.90. To evaluate suicidal ideation, the ninth item of the PHQ-9 was employed. This item, taken from the depression scale, serves as a measure of suicidal ideation (Loerbroks et al., 2016). This approach has been employed in several previous studies (Chow et al., 2018; Mitsui et al., 2018; Nomura et al., 2021). The ninth item of the PHQ-9 inquired about thoughts such as “Thoughts that you would be better off dead or thoughts of hurting yourself in some way?” In this survey, responses were scored as follows: “not at all” (0), “several days,” “more than half of days,” or “almost every day” (1). A score of 1 was considered indicative of suicidal ideation.

### Statistical analysis

First, descriptive statistics were calculated for each variable. Subsequently, a single regression analysis was performed to examine the relationship between suicidal ideation and each variable. Afterward, multicollinearity between variables was assessed, and logistic regression analysis was conducted using the forced entry method to explore the association between suicidal ideation and material deprivation among Vietnamese technical interns in Japan. Confounding factors for the logistic regression analysis were determined based on relevant previous studies, considering participant characteristics and socioeconomic status (Hovey, 2000a,b; Beutel et al., 2016; Fortuna et al., 2016).

Logistic regression analysis was performed with suicidal ideation as the dependent variable and age, gender, length of residence in Japan, Japanese language proficiency, educational background, treatment history, change in monthly income, ownership of a health advisor, and experiences of material deprivation as the independent variables. In the logistic regression analysis, the level of Japanese language proficiency was dichotomized into two categories: fluent Japanese (0) and less fluent Japanese (1). Similarly, the change in monthly income was transformed into a binary variable: no change or increase (0) and decrease (1). Material deprivation variables were also converted into binary values, with 0 indicating no deprivation and 1 indicating experiencing deprivation for a few days, more than half a day, or almost every day. However, for the gas and electricity bills, which are often paid together in Japan, a combined variable was created. It was scored as 1 if the respondent was unable to pay either the gas or electricity bill and 0 otherwise. A total of six material deprivation items were included in the logistic regression analysis. Statistical analyses were conducted using SPSS version 19 software (IBM Corp., Armonk, NY, United States), and a level of *p* < 0.05 (two-sided tests) was considered statistically significant.

## Results

The participants had a mean age of 26.0 ± 5.1 years, with a mean length of residence in Japan of 2.9 ± 4.4 years. On average, they lived with 3.9 ± 2.7 people. Out of the participants, 124 (62.0%) were male and 76 (38.0%) were female. Most participants had a high school education (112 [56.0%]), followed by technical school (47 [23.5%]). A total of 167 (83.5%) participants were single. In terms of Japanese language proficiency, 10 (5.0%) participants could speak fluent Japanese, 83 (41.5%) could speak enough Japanese to not affect their work or study, 81 (40.5%) could speak enough Japanese to not affect their daily life, and 26 (13.0%) could barely speak Japanese. Additionally, 41 (20.5%) participants had pre-existing health conditions. Among the respondents, 68 (34.0%) reported having someone they could consult regarding their health.

Regarding changes in monthly income during the COVID-19 pandemic, 71 (35.5%) participants reported a significant decrease in income of 40% or more, 91 (45.5%) participants reported a slight decrease of 10–40%, 35 (17.5%) reported a change of less than 10%, and three (1.5%) participants reported a slight increase of 10–40%. As for employment status during the COVID-19 pandemic, 15 (7.5%) participants reported dismissal or unemployment. Nine (4.5%) participants reported a change in employment status, 19 (9.5%) participants reported a change in the type (content) of work, and 142 (71.0%) participants reported a reduction in working hours and days. Regarding suicidal ideation, a total of 46 participants (23.0%) scored 1 or more points on the nine items of the PHQ-9, indicating the presence of suicidal ideation (Table 1).

**Table 1:** Characteristics, social-economic situation and suicidal ideation of Vietnam technical interns in Japan (*n* = 200).

Items		*n*	%
Age	Mean ± SD	26.0 ± 5.1	
Duration of residence in Japan	Mean ± SD	2.9 ± 4.4	
Gender	Men	124	62.0
	Women	76	38.0
Educational background	Junior high school and above	2	1.0
	High school	112	56.0
	Technical school	47	23.5
	College	3	1.5
	University	35	17.5
	Graduate school	1	0.5
Japanese language level	Ability to speak fluently	10	5.0
	Ability to speak enough to not affect their work or study	83	41.5
	Ability to speak enough to not affect their daily life	81	40.5
	Barely able to speak Japanese	26	13.0
Pre-existing health conditions	Under treatment or already treated	41	20.5
	No treatment	155	77.5
Having own health advisor	Have	68	34.0
	Do not have	132	66.0
Changes in monthly income under the COVID-19 pandemic	Significant decrease of 40% or more	71	35.5
	Slight decrease/10–40% decrease	91	45.5
	Almost the same	35	17.5
	Slight increase/10–40% increase	3	1.5
The ninth item of the PHQ-9	No suicide-related ideation (0 points)	154	77.0
	Having suicide-related ideation (1–3 points)	46	23.0

**Figure 1:**
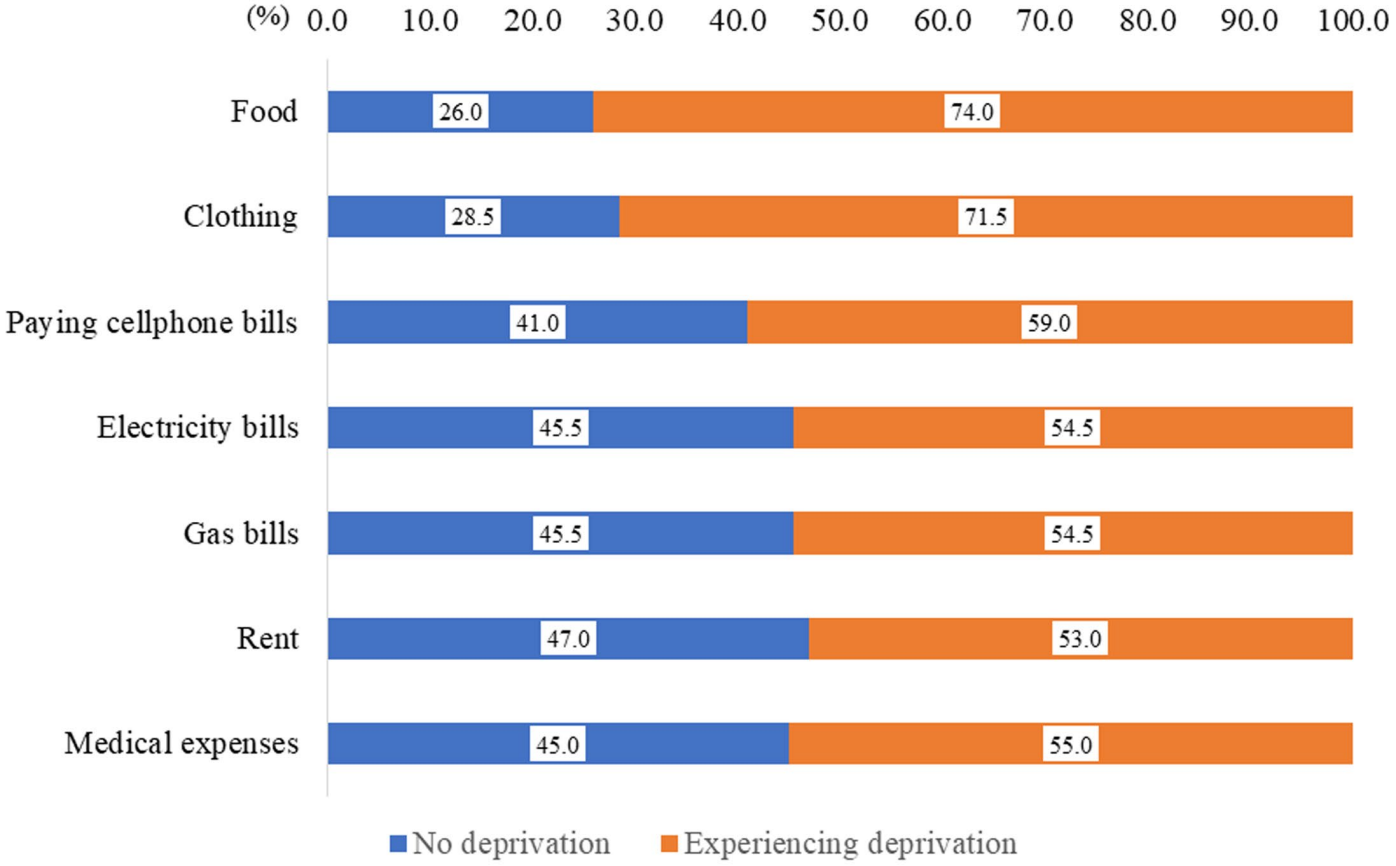
Material deprivation status among Vietnamese technical interns in Japan in 2021.

The frequencies of material deprivation were as follows: for food, 52 participants (26.0%) experienced “No deprivation,” and 148 participants (74.0%) experienced “Deprivation.” Regarding clothing, 57 participants (28.5%) reported “No deprivation,” while 143 participants (71.5%) reported “Deprivation.” For paying cellphone bills, 82 participants (41.0%) experienced “No deprivation,” and 118 participants (59.0%) experienced “Deprivation.” Electricity bills were marked by 91 participants (45.5%) with “No deprivation” and 109 participants (54.5%) with “Deprivation.” Gas bills had 91 participants (45.5%) reporting “No deprivation” and 109 participants (54.5%) reporting “Deprivation.” Concerning rent, 94 participants (47.0%) reported “No deprivation,” while 106 participants (53.0%) reported “Deprivation.” Medical expenses were reported as payable by 90 respondents (45.0%) and unpayable by 110 respondents (55.0%) (Figure 1).

The results of the single regression analysis revealed significant associations between suicidal ideation and three factors: age (*p* = 0.016, odds ratio [OR] = 0.890, 95% confidence interval [CI] = 0.810–0.978), food deprivation (*p* = 0.001, OR = 3.251, 95% CI = 1.639–6.448), and deprivation related to cellphone bills (*p* = 0.003, OR = 2.909, 95% CI = 1.431–5.914). The multivariable logistic regression analysis also showed that age (*p* = 0.031, OR = 0.889, 95% CI = 0.799–0.990), food deprivation (*p* = 0.003, OR = 3.897, 95% CI = 1.597–9.511), and deprivation related to cellphone bills (*p* = 0.021, OR = 3.671, 95% CI = 1.217–11.075) remained significantly associated with suicidal ideation (Table 2).

**Table 2:** Factors related to suicidal ideation of Vietnamese technical interns in Japan.

		Odds ratio [95% CI]	Beta	*p*
Gender		0.614 [0.282, 1.337]	−0.488	0.219
Length of residence in Japan		0.986 [0.787, 1.236]	−0.014	0.905
Age		0.889 [0.799, 0.990]	−0.117	0.031
Japanese language proficiency		0.676 [0.307, 1.491]	−0.391	0.332
Educational background	Junior College/University/Graduate School			0.630
	Technical school	1.873 [0.520, 6.753]	0.628	0.337
	High school and below	1.449 [0.456, 4.602]	0.371	0.529
Treatment history		1.101 [0.457, 2.656]	0.096	0.830
Change in monthly income		2.730 [0.813, 9.163]	1.004	0.104
Having own health advisor		0.982 [0.428, 2.254]	−0.018	0.965
Experiences of material deprivation	Food expenses	3.897 [1.597, 9.511]	1.360	0.003
	Cost of purchasing clothing	0.708 [0.283, 1.771]	−0.345	0.460
	Cellphone bill	3.671 [1.217, 11.075]	1.301	0.021
	Electricity and gas bills	0.529 [0.079, 3.526]	−0.638	0.510
	Rent	2.152 [0.419, 11.059]	0.766	0.359
	Medical expenses	0.423 [0.103, 1.734]	−0.860	0.232

## Discussion

The survey conducted in late 2021 revealed that Vietnamese trainees residing in Japan faced challenges in affording essential items such as food, clothing, cellphones, electricity, gas, and rent. The survey also found that approximately 20% of the trainees showed signs of high suicidal ideation, indicating significant psychological distress among this group. Factors associated with suicidal ideation were age, food deprivation, and inability to pay cellphone bills. The inability to meet cellphone expenses may have contributed to increased isolation among Vietnamese trainees, particularly during the ongoing COVID-19 pandemic. It is crucial to establish a system for early detection and support of trainees experiencing suicidal ideation, along with providing financial assistance and preventing their social isolation.

The COVID-19 pandemic has intensified the economic burdens on Vietnamese technical intern trainees in Japan, impeding their socioeconomic activities and posing a risk of losing residency status and facing impoverishment through dismissals. In response to this, the Japanese Ministry of Health, Labour and Welfare implemented measures to prevent the loss of technical intern trainees’ status of residence (Immigration Services Agency of Japan, n.d.). A report from the Japan Catholic Committee for Refugees, Migrants, and Migrants (Catholic Commission of Japan for Migrants, Refugees, and People on the Move, n.d.), based on consultations with technical intern trainees, disclosed a significant reduction in available work, lowered wages, and a lack of accessible healthcare support for interns with serious health concerns due to the pandemic (Catholic commission of Japan for migrants, refugees, and people on the move, 2020). The prolonged effects of the pandemic have heightened the socioeconomic challenges and anxiety faced by these trainees.

A systematic review and meta-analysis of studies up to 2020 revealed a higher prevalence of suicide, particularly suicidal ideation, and suicide attempts among immigrants (Amiri, 2022). The health of young technical interns in Japan, who are unfamiliar with life in a foreign country, can be significantly impacted by new coronavirus infections. An online survey conducted from January to March 2021, involving 589 Vietnamese residents in Japan, revealed that 37.2% of technical interns experienced symptoms of depression, as indicated by Center for Epidemiologic Studies Depression Scale scores of 16 or higher (Thi et al., 2022). A survey conducted in October 2021, involving 3,262 Japanese technical intern trainee supervisory organizations, reported that over half of the organizations faced challenges in addressing consultations related to mental health issues among technical intern trainees (Ishimaru et al., 2023). In fact, the repeated declarations of a state of emergency in Japan since 2020, due to the COVID-19 pandemic, may have further heightened anxiety among young Vietnamese technical intern trainees, who are far from their home countries and engaged in social activities. Comparing the results of a survey conducted using the same scale among Japanese university students in May–June 2020, the rate of suicidal ideation among Vietnamese technical intern trainees was found to be higher than the 6.7% observed among Japanese individuals (Nomura et al., 2021). Additionally, a cohort study of Japanese workers in 2020 reported an increase in suicidal ideation among the Japanese (Sasaki et al., 2021). This suggests that the outbreak of the novel coronavirus has placed a heavier psychological burden on young Vietnamese technical interns compared to Japanese university students who are accustomed to living in Japan, although this is not a simple comparison.

The results of the logistic regression analysis also found that material deprivation among Vietnamese trainees contributed to suicidal ideation without being influenced by confounding factors. A panel study conducted in South Korea using data from 2012 to 2018 demonstrated a similar association between relative income deprivation and the likelihood of suicide attempts (Pak and Choung, 2020). Moreover, the items associated with suicidal ideation among Vietnamese technical intern trainees in this study were not only related to the experience of lacking essential food for daily life in Japan but also to the experience of deprivation regarding cellphones. Several theories exist regarding the connection between suicidal ideation and cellphones. One theory suggests that excessive cellphone use contributes to isolation among young individuals, thus increasing the risk of suicidal ideation (Huang et al., 2022; Li et al., 2022). Another theory proposes that cellphones have reduced isolation and improved mental health among young people, especially during the COVID-19 pandemic. Supporting studies include a 2022 survey of Japanese university students, which found that smartphone use reduced the risk of loneliness during the pandemic (Nguyen et al., 2022), a study conducted in Belgium during the pandemic that demonstrated individuals who felt lonely were more likely to use social media to cope with social isolation,(Cauberghe et al., 2021) and a study conducted in Australia during the pandemic that reported lower levels of adolescent loneliness among those who used technology to connect with society (Li et al., 2021).

Therefore, during the unprecedented circumstances of the COVID-19 pandemic, it is possible that young individuals did not rely solely on social media but rather utilized it to alleviate loneliness. This may also be applicable to Vietnamese technical interns who have relocated from their home country to Japan. Considering that most trainees in the current study were in their early 20s, it is likely that many of them maintained contact with family members and distant friends in their home countries through social media during the initial stages of the COVID-19 outbreak. Cellphones were likely an essential tool for technical interns to stay connected with their loved ones and seek emotional support during this challenging time. The study findings emphasize that deprivation of this crucial tool had a significant impact on the participants’ mental well-being.

To prevent suicidal ideation among Vietnamese technical intern trainees in Japan, it is crucial to offer social and economic support to prevent material deprivation. It is equally important to consistently monitor the mental health status of Vietnamese technical intern trainees, even beyond the conclusion of the COVID-19 pandemic, to better prepare for future pandemics. By implementing these measures, the well-being and overall stability of Vietnamese technical intern trainees can be safeguarded.

The current study has several limitations that should be acknowledged. Firstly, the sample size was small. Secondly, the assessment of material deprivation and suicide-related ideation relied on self-reported measures, which could be subject to cognitive bias. Thirdly, the recruitment of participants through Facebook may have introduced selection bias, as it may have attracted individuals with a higher interest in the topic of the study. Additionally, the study did not specifically account for the emotional stress experienced by Vietnamese trainees living away from their home country. Lastly, since the data were collected after the onset of the COVID-19 pandemic, it was not possible to compare them with pre-pandemic data. Despite these limitations, the survey offers valuable clinical insights into suicidal ideation among Vietnamese technical interns in Japan and provides scientific data on the effects of material deprivation during the COVID-19 pandemic.

## Conclusion

The purpose of this study was to examine the deprivation and suicidal ideation of Vietnamese technical intern trainees during the COVID-19 pandemic. The findings revealed that Vietnamese technical intern trainees in Japan faced various forms of material deprivation. Moreover, a significant proportion (23.0%) of these trainees reported experiencing suicidal ideation, indicating a high prevalence of severe psychological issues. Contributing factors included food deprivation, and inability to pay cellphone bills. Lack of food contributes to poor mental health, and the inability to cover cellphone expenses may increase isolation among Vietnamese trainees in Japan during the ongoing pandemic. Providing financial assistance is crucial to preventing mental health problems among Vietnamese technical intern trainees.

## Data availability statement

The datasets presented in this article are not readily available because the datasets analyzed in the current study are available from the corresponding author on reasonable request. Requests to access the datasets should be directed to TY, yamashita@tr.kobe-ccn.ac.jp.

## Ethics statement

The studies involving humans were approved by the Kobe City College of Nursing Ethics Research Committee (approval number: 20124-05). The studies were conducted in accordance with the local legislation and institutional requirements. The participants provided their written informed consent to participate in this study.

## Author contributions

TY conceived the original idea for the study. TY, PQ, and KK designed the questionnaire, analyzed the data, and wrote the first draft of the manuscript. PQ, CY, EN, ES-S, SI, and KK contributed to the interpretation of the results. All authors made substantial intellectual contributions to the study and approved the final draft of the manuscript.
